# Women's wheelchair basketball lineup analysis at the Tokyo 2020 paralympic games: game related statistics explaining team sport performance

**DOI:** 10.3389/fspor.2023.1281865

**Published:** 2023-11-06

**Authors:** William Becerra-Muñoz, Jiahui Wang, Javier Pérez-Tejero

**Affiliations:** ^1^ “Sanitas Foundation” Chair for Inclusive Sport Studies, Health and Human Performance Department, Faculty of Physical Activity and Sport Sciences-INEF, AFIPE Research Group, Universidad Politécnica de Madrid, Madrid, Spain; ^2^ Faculty of Movement and Rehabilitation Science, Katholic University Leuven, Leuven, Belgium

**Keywords:** game statistics, disability sport, paralympic sport, team performance, team sport, lineup analysis

## Abstract

**Introduction:**

Performance analysis through game-related statistics in wheelchair basketball (WB) has focused mainly on the study of the individual efficiency of players according to their functional classification. However, there is little evidence focusing on lineup performances (five players on court) and their composition. Thus, the objective of present study was to analyze the efficiency of the women's WB lineups used during the Tokyo 2020 Paralympic Games (PG) and to determine the variables that best discriminated the lineup performances according to the final point differential.

**Methods:**

The sample comprised 507 lineups used in the 31 games by the 10 national teams during the competition. Fifty-one different lineup types (LTs) were categorized. A discriminant analysis was carried out to compare the lineups with a positive and negative point difference according to the game type (balanced and unbalanced games).

**Results:**

It was found that LTs 16 (1-1.5-2.5-4-4.5), 47 (1-2-2.5-4-4.5) and 14 (1-1.5-2.5-4.5-4.5) had the best means of efficiency in field goals (LT 16 = 52%; LT 47 = 44% and LT = 40%), while LT 50 (1-2-3-4-4) obtained the highest mean difference in points (3.67 ± 10.67). The variables that best discriminated winner teams in balanced games were field goal efficiency (SC = 0.55), assists (SC = 0.50) and turnovers (SC = −0.41).

**Discussion:**

Field goal efficiency, assists, turnovers and steals are the game-related statistics most associated with the success of a lineup used in balanced games in WB in PG competition; this could be taken into account by coaches when deciding how to compose a given lineup in a moment of the game, to adequately select players from different functional classifications for the final squad and to choose training content related to the indicated game-related statistics, as they explain success at this competition level.

## 1. Introduction

Wheelchair basketball (WB) is a sport of high intensity and dynamic game rhythm, which enjoys great popularity within the Paralympic Games (PG). In addition, WB is one of the sports most practiced by people with physical disabilities (1). It demands a good physical condition, technical expertise, teamwork and complex decision-making at elite level (2–4). Most of its characteristics and rules are very similar to running basketball; however, the use of the wheelchair and the functional classification system are the main differences. For the competition, each player is classified into a functional class (FC) ranging from 1.0 to 4.5 according to their functional movement capacity (8 classes, differences of 0.5 between classes according to trunk control, function of upper and lower limbs) to ensure that the player is capable of carrying out fundamental technical actions, such as pushing the chair, braking, turning (5), dribbling, moving forward and shooting to the basket. The higher the functional class, the greater the player's functional ability to move. This, to a large extent, conditions the role and actions that the player executes on the court. The total sum of the FC of the five players on court must not exceed 14 points at international competitions (6). Due to its importance, the studies that analyze factors influencing sport performance in WB have increased considerably in recent years, mainly addressing aspects related to biomechanical analysis and individual player performance and, to a lesser extent, the analysis of team performance and its efficacy during competition (2, 4, 7, 8).

Thus, in the analysis of physical performance, research such as that of Granados et al. (3), Gil et al. (9) and Romarate et al. (10) assessed to different level WB players in pick-up test, maximal pass test, agility T-test and medicine ball throw, where identified that capacities such as power, arm strength, agility and stamina are decisive. Other authors have evaluated WB players in tests with and without the ball, thus determining physical performance profiles for the different FCs (11–14). The analysis of technical skills in WB has also been a field of considerable interest in the studies that have been carried out: The kinematic patterns of free throws have been analyzed to determine the individual technique adopted by each player based on their FC, their posture and their support in the wheelchair (15). Also, studies such as those by Limroongreungrat et al. (16), Bergamini et al. (17), Chénier et al. (18) and Wang et al. (1) have extensively studied how the fundamental technical actions (dribbling, driving the chair, passing and shooting) and the ranges of movement of the upper limbs influence player performance.

On the other hand, several studies have shown that there are differences in performance related game statistics between players of different FCs (19–22). Some of them analyzed differences in players’ performance related to the game statistics during competition, comparing the interaction of FC and game position (22), FC and final team ranking (20), and FC and game time (23) with performance in field goals, free throws, assists, offensive and defensive rebounds, turnovers and steals, finding significant differences among all functional classes; however, these differences are appreciated the greater the distance is between functional classes, with no significant differences found between adjacent FCs.

In the analysis of team performance, Gómez et al. (19) analyzed the statistics related to the teams’ play during men's and women's competitions at the international level and they identified the variables that best discriminated between winning and losing teams according to the game type (balanced and unbalanced). In addition, they assessed whether the quality of the opponent and the four basketball performance factors (field goal efficiency, turnover ratio, offensive rebound percentage and free throw efficiency) (24, 25) could predict the final difference in points by gender and type of game. Thus, they found that 2-point field goals scored, free throws, assists, and fouls received were the most determining factors in the men's competition, while 2-point field goals scored was the most determining factor in the women's competition. In this regard and in running basketball, the most important factors that discriminate the final result of a game (winner–loser) have also been analyzed; for example, Canuto and de Almeida (26) carried out a systematic review with meta-analysis on this issue, which indicated that, in line with the WB literature, field goal efficacy and assists, in addition to defensive rebounds, are the factors that best discriminate the final result of a game (winner–loser) in competition, taking into account the quality of the opponent and the phase of the competition.

Based on different studies that have been carried out in WB, it could be said that FC is a fundamental aspect to take into account when analyzing WB performance, as players with different functional class present differences in volume of action (6), physical performance (27–29) and playing role (21, 30). In this regard, the coach must take this aspect into account when choosing the players that will make up the final squad for a given competition: whom he/she must align in each game and situation in order to promote the best possible interactions between players and FCs so as to maximize collective performance in that specific competition, game and moment (31). However, even though it seems important to ask what kind of lineup type it is possible to configure according to the 12 (number of players per team) FCs of the players’ team, what the prevalence is of a given lineup use in a given WB competition and what the game statistics are that indicate greater efficiency between one given lineup or another, there are very few studies following this direction.

In running basketball, not too many studies have been carried out analyzing the influence of different lineups on team performance. Sandri et al. (32) carried out an analysis of the influence of relationships between teammates on the court on the shooting performance index, finding differences in the individual performance of the player when accompanied by different combinations of teammates on the court. Clay and Clay (33) examined the impact of the use and depth of bench players on team performance and success, finding that a high rotation (multiple lineups) of players generates advantages, especially in defensive efficiency, while managing a short rotation (few lineups) can generate advantages in shooting percentages, ball control and other offensive performance variables. Other investigations have aimed at analyzing how the rotations and the different possible lineups on the field influence the offensive and defensive team performance, considering the role of the players (34) and their anthropometric characteristics (35).

In WB, García-Fresneda (36) analyzed the behavior and efficiency of the different lineups at the men's WB World Championship in 2014, categorizing the different lineups into types according to the predominance of low (1.0–1.5), medium (2-3.5) and high (4–4.5) FC of the players on the roster. He found that the most prevalent lineups in that competition were type D (two low, one medium and two high), E (one low, two medium and two high) and C (three or more medium). In addition, he indicated that there were no significant differences between offensive and defensive tactical behavior, observing a predominance of positional offense and zone defense in the three types of lineups. There are multiple possibilities in the composition of the WB lineups, conditioned by the availability of players from different FCs in the team. However, there is not enough evidence regarding different LTs performance, analyzed through game statistics, to determine which are the most used according to the type of game and the phase of the competition. Additionally, the scarcity of studies in the female population in WB competition is noteworthy.

For all above, the aim of this study was twofold: firstly, to analyze the most used lineups in the women's WB competition at the Tokyo 2020 PG according to their game statistics (to determine which lineups seem to be the most efficient in balanced games). Secondly, to identify the game statistics that best discriminate between lineups with a positive and negative final point differential for balanced and unbalanced games.

## 2. Material and method

### 2.1. Sample

**Figure 1:**
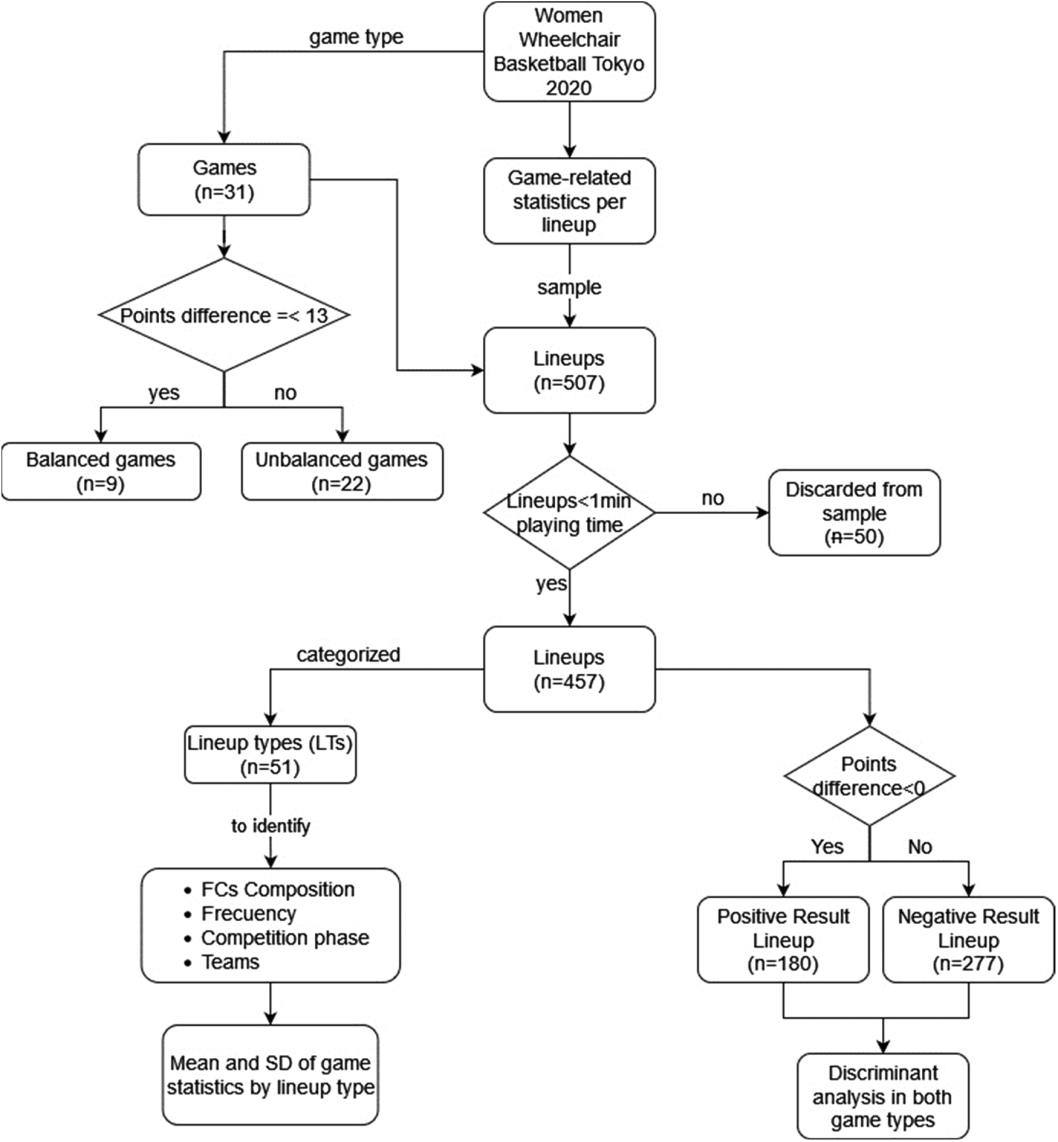
Flow chart of procedure.

The official game statistics for the women's WB competition at the Tokyo 2020 PG were obtained from the official website: the “Line-Up Analysis” report concretely. The sample consisted of the 507 lineups used in 31 games played by the 10 national teams during the different phases of the competition: group phase (round robin per group) and playoff phase (quarterfinals, semifinals and final). The game-related statistics gathered were field goals scored (FGS) and received (FGR) (both successful and attempted), offensive rebounds (ORs), defensive rebounds (DRs), assists (ASs), turnovers (TOs) and steals (STs) both for and against. Fifty-one lineup types (LTs, combination of five players on court for a given team) were identified and categorized (from those 507 lineups), identifying their total frequency, number of games where they were used, phases of the competition in which each was used and players that made up each lineup according to their FC, and finally, distinguishing the different national teams that used a specific LT throughout the competition (Figure 1).

By criteria, lineups that had a value of less than one minute in playing time were not taken into account (24), thus leaving a final sample of 457 lineups for subsequent analysis. All the variables were normalized, taking into consideration the proposal for the normalization of the game statistics of each lineup by playing time (25) and efficacy percentages for TCs, ROs and RDs were calculated for each alignment (24). A k-means cluster was carried out to classify the games, depending final result, by point differential, but because in some games there was a great difference in points between the two teams at the end, the cut-off value obtained that separates the clusters was very high, classifying as even those games with differences greater than 45 points. Therefore, the cut-off point used by Gómez et al. (19), was considered. This classifies games ranging from 1 to 13 points as balanced games (9 games) and games with differences greater than 13 points as unbalanced games (22 games). The values of the 457 lineups were classified into two groups: those with a point difference (plus/minus) above 0 as a positive result (180 lineups) and those with differences equal to or less than 0 as a negative result (277 lineups) (Figure 1).

### 2.2. Statistical analysis

For the first aim, descriptive statistics were obtained for the variables calculated from game-related statistics and the four basketball performance factors. The value of the mean (M), the standard deviation (SD), the maximum value and the minimum value of the given game-related statistic of each LT that was used by the national teams for balanced games were taken into account. For the second aim, a discriminant analysis was carried out to identify the variables that best classify the lineups with a positive/negative final result of points in balanced and unbalanced games. Structural coefficients (SC) above │0.30│ made it possible to identify the variables that best contribute to differentiating LTs with a positive result from those that had a negative result (37). Validation of the discriminant models was performed using an exclusion classification. Cross-validation of the discriminant models was performed using the “leave-one-out” classification (38). The statistical analysis was carried out through Excel 2019 (Microsoft. Redmond, WA, United States, 2019) and IBM SPSS Statistics version 29 (IBM. Armonk, NY, United States, 2022). The significance level was set at *p* < 0.05.

## 3. Results

### 3.1. Descriptive analysis of the alignments

The 118 female players participating in the WB Tokyo 2020 PG competition by team, according to their FC, are indicated in Table 1. All national teams had 12 players for the competition, except for Algeria and Canada, who had 11 players in their squad. It can be seen that Netherlands was the only team that had at least one player for each functional class in its squad, unlike Australia, which only had players from five different FCs, with six class 1.0 players. On the other hand, teams like Algeria and Great Britain had up to four 4.0-point players available in their roster, while Canada and Germany had the same number for 4.5-point players. The functional classes that had the greatest presence of players in the competition were 1.0 (23 players), followed by 4.0 and 4.5 (20 players of each).

**Table 1:** Players’ functional classification by national teams at the 2020 Tokyo PG WB female competition.

** **	Functional Class								
Team	**P1** **.** **0**	**P1** **.** **5**	**P2** **.** **0**	**P2** **.** **5**	**P3** **.** **0**	**P3** **.** **5**	**P4** **.** **0**	**P4** **.** **5**	**TP**
Netherlands	2	2	1	2	1	1	2	1	12
China	2	2	2	0	1	0	3	2	12
United States	2	2	1	2	0	2	1	2	12
Germany	2	1	2	2	0	1	0	4	12
Canada	2	2	0	1	1	1	0	4	11
Japan	2	1	1	3	1	0	2	2	12
Great Britain	2	1	0	2	1	1	4	1	12
Spain	2	0	3	1	2	0	3	1	12
Australia	6	0	0	2	1	0	1	2	12
Algeria	1	2	0	2	1	0	4	1	11
Total	23	13	10	17	9	6	20	20	118

TP, Summary of players per team; P1.0-4.5, players with functional class 1.0-4.5.

Table 2 shows each LT used during the competition, the FCs that compose it, the number of times the LT was used during the competition, the phases of the competition in which it was used, the number of games in which it was used and the teams that used the given LT. Of the 47 different LTs categorized in the competition, all were used during the round robin phase, 24 LTs during the qualifying playoffs, 24 LTs in the quarterfinals, 8 LTs in the semifinals, 7 LTs in the bronze medal game, and 5 LTs in the gold medal final. The average LT number used during the competition was 7.5 per game. The highest number of LTs used was in games 19 and 53 (13 LTs). In games 2, 21 and 64 the lowest LT values used (3 LT) were found. Thus, the lineup that was repeated the most times during the competition was LT 14 (1-1.5-2.5-4.5-4.5), followed by LT 29 (1-1-2.5-4.5-4.5), LT 16 (1 −1.5-2.5-4-4.5) and LT 23 (1-1.5-3-4-4.5), the latter obtained the highest values in the number of games (18) and national teams that used it (5).

**Table 2:** Lineup types (LTs) used at the 2020 Tokyo PG WB female competition.

LT	Functional classes	F	Competition phase	G	Teams
14	1-1.5-2.5-4.5-4.5	68	RR-5/6°-QF-SF-3/4°	15	CAN-GER-JPN
29*	1-1-2.5-4.5-4.5	37	RR-9/10°-QF-3/4°	8	AUS-GER
16*	1-1.5-2.5-4-4.5	36	RR-9/10°-5/6°-QF-SF-F	15	ALG-JPN-NED
23	1-1.5-3-4-4.5	34	RR-5/6°-QF-SF-F	18	ALG-CHN-GBR-JPN-NED
47	1-2-2.5-4-4.5	34	RR-7/8°-5/6°-QF	14	SPA-JPN-NED-USA
50	1-2-3-4-4	27	RR-7/8°-QF-SF-F	12	CHN-SPA-NED
21*	1-1.5-3-3.5-4.5	25	RR-5/6°-QF-F	8	CAN-NED
43*	1-2-2.5-3.5-4.5	24	RR-QF-SF-3/4°	12	GER-NED-USA
30	1-1-2.5-4-4.5	22	RR-9/10°	5	AUS
39	1-2.5-2.5-4-4	22	RR-QF-7/8°	7	GBR-NED
34	1-1-3-4.5-4.5	16	RR-9/10°-5/6°-QF	9	AUS-CAN
15	1-1.5-2.5-4-4	13	RR-9/10°	5	ALG
32	1-1-3.5-4-4	13	RR-QF-7/8°	5	GBR
18	1-1.5-2-4.5-4.5	12	RR-QF-SF-3/4°	7	GER
37*	1-2.5-2.5-3.5-4	11	RR-QF-7/8°	7	GBR-NED
35*	1-1-3-4-4.5	8	RR-9/10°	5	AUS-CHN
40	1-2.5-3-3.5-4	8	RR-7/8°-QF-F	7	GBR-NED
9	1.5-2-2.5-4-4	7	RR-QF	4	JPN-NED
28	1-1-2.5-3-4.5	7	RR	3	AUS
31	1-1-2-4.5-4.5	7	RR-QF	3	GER
20	1-1.5-3.5-4-4	6	RR-QF	4	NED
33	1-1-3.5-4-4.5	6	RR-QF-SF	6	GBR-NED-USA
36	1-1-4-4-4	6	RR-QF-7/8°	4	GBR-SPA
38	1-2.5-2.5-3.5-4.5	6	RR-7/8°-SF-3/4°	5	USA-NED-GBR
42	1-2.5-3-3-4.5	6	RR-7/8°-QF	5	SPA
8	1.5-2-2.5-3.5-4.5	5	RR-QF-3/4°	4	NED-USA
22*	1-1.5-3-4-4	5	RR-9/10°-7/8°	4	ALG-GBR
27*	1-1-2.5-3-4	5	RR-9/10°	4	AUS-SPA

LT, lineup type; F, frequency; G, games; RR, round robin; QF, quarter final game; 9/10°, 9° place game; 7/8°; 7° place game; 5/6°, 5° place game; SF, semifinal game; 3/4°, bronze medal game; F, final game.

* Less than 14 points. CAN, Canada; GER, Germany; JPN, Japan; AUS, Australia; ALG, Algeria; NED, Netherlands; CHN, China; GBR, Great Britain; SPA, Spain; USA, United States.

In relation to the frequency analysis by competition phase, it was observed that LT 14 (1-1.5-2.5-4.5-4.5) was the one that was repeated the most times in each of the phases of the competition, during the round robin phase it was repeated 38 times, followed by LT 29 (1-1-2.5-4.5-4.5; 27 times) and LT 16 (1-1.5-2.5-4-4.5, 23 times). During the playoffs phase, for the quarterfinals LT 14 (1-1.5-2.5-4.5-4.5) was used 16 times, followed by LT 47 (1-2-2.5-4-4.5; 11 times) and LT 16 (1-1.5-2.5-4-4.5, 10 times). During the semifinals and games for the bronze medal and for the gold medal, LT 14 (1-1.5-2.5-4.5-4.5) was used 8 times, followed by LT 43 (1-2-2.5-3.5-4.5; 5 times), LT 18 (1-1.5-2-4.5-4.5, 4 times) and LT 23 (1-1.5-3-4-4.5, 4 times). Of the 31 games played, 9 had point differences of less than 13 (balanced games; six during the round robin phase, the two semifinals and the bronze medal game); while the gold medal game resulted in a 19-points difference game.

The national teams used an average of 8.6 LTs during the whole competition, with Netherlands being the team that used the most LTs (18), followed by Great Britain (10) and Spain (10). In contrast, Japan was the team that used the least LTs during the competition (5), followed by China (6) and Canada (6). The descriptors for the game-related statistics in balanced games are presented in Table 3: during the competition phases, LT 14 (1-1.5-2.5-4.5-4.5) was the most used in balanced games, followed by LT 43 (1-2-2.5-3.5-4.5), LT 18 (1-1.5-2-4.5-4.5) and LT 23 (1-1.5-3-4-4.5), respectively. LT 50 (1-2-3-4-4), used by China, had the highest value in playing time (40 min) during the semifinal game against the United States, as well as the highest average (24.36 min ±13.72), followed by LT 23 (15.07 min ±9.05) and LT 43 (15.13 min ±14.61), all of them regarding playing time.

**Table 3:** Descriptive statistics of lineup types in balanced games at the 2020 Tokyo PG WB female competition.

LT	F	Teams		Min	Plus/Minus	%FG	%OR	%DR	AS	TO	ST
14	22	CAN-GER-JPN	M	5.04	−1.30	39.9	15.5	76.1	12.32	19.64	4.45
			SD	4.40	3.96	14.9	17.9	20.4	15.02	8.82	7.59
43	10	USA	M	15.13	−0.30	37.4	10.5	88.4	7.27	17.92	3.85
			SD	14.61	6.99	24.3	12.9	11.3	5.43	13.90	6.01
18	8	GER	M	7.53	−0.63	32.3	14.7	83.4	12.06	13.23	2.44
			SD	7.01	5.66	19.9	14.6	19.0	10.99	10.98	4.06
23	8	CHN-JPN-NED	M	15.07	0.88	37.7	17.3	79.8	9.43	15.85	3.96
			SD	9.05	6.83	7.9	13.0	13.7	6.94	7.09	5.13
16	7	JPN-NED	M	8.24	2.00	52.3	16.2	79.5	8.77	16.55	2.16
			SD	6.03	3.96	25.4	21.8	17.6	13.03	13.40	2.89
21	6	NED-CAN	M	3.61	1.17	24.1	45.0	70.0	16.24	10.40	5.56
			SD	1.92	3.37	28.5	46.4	34.6	18.89	11.43	8.86
47	6	NED-JPN	M	4.91	2.17	43.8	22.7	83.2	9.23	23.87	4.40
			SD	3.25	4.40	22.0	18.6	18.3	16.49	20.28	8.81
39	4	GBR	M	6.60	1.00	34.2	18.8	87.5	10.04	18.05	9.74
			SD	3.49	2.58	4.2	14.2	16.0	3.38	7.14	12.58
50	3	CNH	M	24.36	3.67	29.4	16.7	89.7	9.06	14.08	3.90
			SD	13.72	10.07	16.7	10.4	8.7	3.45	2.95	1.73

LT, lineup type; F, frequency; M, mean; SD, standard deviation; Plus/Minus, point difference result; FG, field goals efficiency; OR, offensive rebounds; DR, defensive rebounds; AS, assists; TO, turnovers; ST, steals.

On the other hand, LT 50 (1-2-3-4-4) was used only by China (silver medal) in balanced games, obtained the highest average in the difference of points scored and received by lineup (plus/minus), followed by the LT 47 (1-2- 2.5-4-4.5) and LT 16 (1-1.5-2.5-4-4.5), the latter two used by Netherlands (gold medal) and Japan. For field goals efficiency (%FG), LT 16 obtained the highest value (100%) when it was used by Japan in game 39 (only two minutes of play and two field goals taken and scored), followed by LT 43 (1-2-2.5-3.5-4.5) and LT 38 (1-2.5-2.5-3.5-4.5) both reaching 83.3%, the latter two used by the United States. However, regarding the mean values for the same variable (%FG), LT 16 (1-1.5-2.5-4-4.5) obtained the highest value (52.3% ±25.4), followed by LT 47 (1-2-2.5-4-4.5; 43.8% ±22) and LT 14 (1-1.5-2.5-4.5-4.5; 39.9% ±14.9).

### 3.2. Lineup discriminant analysis by outcome

Means and standard deviations for the game-related statistics by point differential (plus/minus) related to the lineups used during the competition were assessed. Significant differences can be observed for both types of games: for balanced games, lineups with positive results presented better field goal efficiency, greater number of assists and steals and fewer turnovers. For unbalanced games, positive-scoring lineups had better field goal efficiency, offensive rebounding and defensive rebounding efficiency, more assists and steals, and reporting fewer turnovers, while fewer assists and ball steals from the rival lineup were assessed.

The discriminant analysis differentiated between the lineups with a positive result from those that had a negative result for balanced and unbalanced games (see Table 4). The most decisive variables to discriminate the lineups with positive and negative results in balanced games (*λ*=0.51; CC = 0.70; *p* < 0,001) were field goal efficiency (SC = 0.55), assists (SC = 0.50) and turnovers (SC = −0.41). In unbalanced games (*λ*=0.45; CC = 0.74; *p* < 0,001), field goals efficiency (SC = 0.73), assists (SC = 0.63) and assists from the opponent team (SC = −0.54) were the variables that best discriminate between lineups with positive and negative results. The cross-validation of the discriminant model reported a correct percentage of reclassification of the cases of 80.5% for balanced games and 89% for unbalanced games**.**

**Table 4:** Means, standard deviations, and structural coefficients of lineup game-related statistics with positive and negative results in balanced and unbalanced games.

Game Statistics	Balanced games					Unbalanced games				
	Positive Result		Negative Result		SC	Positive Result		Negative Result		SC
	M	SD	M	SD		M	SD	M	SD	
% FG**	51.27	14.53	31.28	20.39	0.55*	52.36	17.00	22.28	19.45	0.73*
% OR	26.68	25.14	30.47	82.82	−0.03	27.53	26.86	18.61	21.57	0.17
% DR	83.32	14.66	80.03	19.68	0.09	83.38	17.16	71.90	29.01	0.21
AS**	23.28	11.75	13.41	8.76	0.50*	26.62	12.70	10.49	10.44	0.63*
TO**	6.40	5.80	14.41	11.73	−0.41*	9.18	10.38	17.23	15.80	−0.26
ST**	5.92	6.98	2.69	4.60	0.29	7.86	9.57	4.21	7.69	0.19
ASr	15.28	12.49	19.13	11.45	−0.16	8.34	8.75	23.84	15.25	−0.54*
TOr	13.27	14.24	8.58	8.88	0.21	18.64	15.01	10.69	12.26	0.27
STr	2.78	3.87	5.07	7.50	−0,18	3.64	6.56	7.50	9.92	0.20

* Values of the discriminant coefficients ≥│0.30│ (*p* < 0.001).

** Significant differences in balanced games (*p* < 0.05); There are significant differences in all variables in unbalanced games (*p* < 0.05). M, mean; SD, standard deviation; FG, field goals efficiency; OR, offensive rebounds; DR, defensive rebounds; AS, assists; TO, turnovers; ST, steals; ASr, rival assists; Tor, rival turnovers; STr, rival steals.

## 4. Discussion

To the best of our knowledge, there is no such lineup analysis available in the scientific literature regarding top-level female WB competition, with specific considerations for coaches when preparing the team roster (e.g., team configuration and representation of different functional classes) and managing lineups during competition (for example, indicating which game-related statistics explain LT performance in balanced games). In relation to the results obtained, it was relevant to observe that Netherlands, who had at least one player per each FC and was the team that used the largest number of different LTs during the competition (tripling the one that used the least number of LTs, Japan), was the team that won the gold medal. In contrast, Algeria, which had the lowest FC availability among its players, was in last place in the final competition ranking.

In this regard, Clay and Clay (33) highlighted in running basketball the advantage of having depth on the bench so as to have different rotation options and lineups. However, China won the silver medal, without having 2.5- and 3.5-point players on its roster and using a low number of LTs compared to the other teams. Furthermore, two of the six LTs used by China during the competition reported the highest value in points difference (LT 50) and the fourth best in %FG (LT 23). It should be noted that these two LTs were reported in the study by García-Fresneda (36) as the two most used during men's world championship in 2014. The FCs with the greatest presence of players in the competition (1.0-, 4.0- and 4.5-point players) were related to the LTs most used by the national teams, thus predominating in the composition of the lineups: having the presence of two 1.0-point players and two players from 4 to 4.5 points on court. This distribution of the number of players per FC was related to those reported in the 2006 women's world championship by Molik et al. (20).

Seven out of nine LTs most used in balanced games (14, 18, 23, 16, 47, 39 and 50, see Table 2) showed at least two players (one 4.0-point player and one 4.5-point player) in their composition, which seems to indicate that there was a trend during the competition of using players with a higher FC by the teams (thus compensated with the use of 1.0- and 1.5-point players in the same LT). Similarly, seven out of nine LTs used in balanced games (14, 43, 18, 23, 16, 47 and 39) reported values greater than 30% in their %FG, reaching up to 52% for LT 16; these values are similar to those reported by Molik et al. (20) in the 2006 world championships and higher than the 1998 world championships reported by Vanlandewijck et al. (22), where the %FG was not higher than 30%. This seems to indicate that during recent years there has not been a significant change in the female WB performance in terms of field goals efficiency; however, in our study, not having 3- and 2-point field goals from the official lineup analysis report separately made it difficult to conclude this with certainty.

The most used LTs during the competition in balanced games coincide with the three predominant types (C, D and E) reported by García-Fresneda (36), but not the specific composition of the LTs: from the nine reported LTs in that study, two of these (50 and 23) appear as the most used LTs in this current study, with a percentage of 12%, five LTs (18, 16, 21, 47 and 39) with percentages between 0 and 1.5% and two LTs (14 and 43) were not used in the 2014 men's world championship (36). Thus, LT 16 and 47, for example, showed the highest values in point difference and high values in field goals efficiency, being two LTs that have a very similar composition (with only a FC difference in the second player in the lineup (1.5- and 2-point player). In this regard, it should be noted that eight LTs used did not reach the regulatory maximum of 14 points allowed (that is, they used less than 14 points on the court, see Table 2), which can be a tactical criterion (the case of Netherlands with LT 16, 29 and 43) as a derivative of the (lower) LT possibilities, depending on the team roster (see Table 1).

Field goal efficiency variables, assists and turnovers were the game statistics that best discriminated between the total number of lineups used that had a positive or negative result in point difference during balanced games, only coinciding with the discriminant variable of 2-point shots scored (SC = 0.37) reported by Gómez et al. (19) when comparing winning and losing female teams in balanced games at this performance level. This seems to indicate that today the women's competition, contrary to what was considered a few years ago (19), shows a greater diversity of tactics and strategies, given that, in addition to field goals, a greater number of assists (teamwork) and avoiding turnovers were decisive in the result. Likewise, ball steals (SC = 0.29) were also established as a game-related statistic that distinguishes winning teams, highlighting their greater defensive capacity. From the above, it seems that the skills related to handling the ball and passing are essential when it comes to performing at the highest level, avoiding losses as much as possible. In the same way, wheelchair skills to increase the defensive level and increase the losses of the opponents seem to be important at this elite competitive level (1, 20–22). This may lead to training content suggestions for coaches, in order to prioritize these elements when preparing for WB elite competition.

When it comes to unbalanced games, similarly to previous studies (19, 20), field goal efficiency was the most determining variable to explain the success in the final result of the games. Although the FG% was the most decisive variable, it was not the only one. It can be seen that there were differences in favor of LTs with a positive result of almost 50% in the means comparison of the game statistics. It would be interesting to analyze whether, in some of those unbalanced matches, the losing team in the play-by-play used one LT that worked better than another or, despite the rotations, the losing team was always inferior to its rival. Thus, future studies on lineup analysis should take into account when a given LT is capable of generating an unbalanced difference on the scoreboard (at the end of the game, but also after its participation).

One of the limitations of this work was that the game-related statistics for 2 and 3 field goal points and free throws of each LT were not available in the official report. Furthermore, although statistics against were taken into account (e.g., %FG received, rival assists, offensive rebounds against, etc.) these data were not discriminated e.g., by the LT of the rival team. Therefore, it becomes invaluable to identify which LTs performed best against a certain LT of the rival team. This consideration is important for future studies, in line with what was proposed by Francis et al. (31) on the importance of analyzing offensive statistics such as FG% taking into account the defensive actions of the rival team such as the pressure zone and other contextual variables. This is due to the fact that the design has adhered to the information that was available from the official lineup analysis report. In addition, considering the moment of the game in which the different LTs are used (i.e., time series analysis) could have expanded the information reported (39), explaining the changes at a certain moment based on the game situation. For future studies, in addition to trying to solve the previous limitations, it is intended to replicate the study for the male competition at the PG and to observe possible differences in performance. Furthermore, it would be interesting to explore whether there are significant differences in the game-related statistics for the same LTs when they are made up of different players and to analyze the impact of these substitutions during game development (and not only the different results of score differences in the final points): applications of “play by play” data analysis could be of interest in this regard.

## 5. Conclusions

The LT 14 (1-1.5-2.5-4.5-4.5) was the most used lineup throughout the entire competition, with a frequency almost double that of the second most used lineup. However, the LT 23 (1-1.5-3-4-4.5), with fifty percent less use than the previous one, was used by up to five different teams. The team lineups that presented the best efficiency in the game-related statistics in the WB competition at the Tokyo PG female competition for balanced games were the lineup types 16 (1-1.5-2.5-4-4.5), 47 (1-2-2.5-4-4.5), 14 (1-1.5-2.5-4.5-4.5) and 50 (1-2-3-4-4). Moreover, field goal efficiency, assists and avoiding rival assists are more decisive factors in unbalanced games. On the other hand, field goal efficiency, assists, turnovers (avoid them) and steals are the game-related statistics that determine the success of a lineup used in balanced games in this competition. Both conclusions could be taken into account by coaches when deciding how to compose a given lineup in a moment of the game, to adequately select players from different FCs for the final squad and to choose training content related to the indicated game-related statistics as they explain success at this competition level.

## Data Availability

The original contributions presented in the study are included in the article/Supplementary Material, further inquiries can be directed to the corresponding author.
